# Comparison of gemcitabine plus oxaliplatin versus gemcitabine plus nab‐paclitaxel as first‐line chemotherapy for advanced pancreatic adenocarcinoma: A single‐center retrospective analysis

**DOI:** 10.1002/cam4.6334

**Published:** 2023-08-03

**Authors:** Konstantin Schlick, Antonia Gantschnigg, Alexander Seymer, Florian Huemer, Richard Greil, Lukas Weiss

**Affiliations:** ^1^ Department of Internal Medicine III with Haematology, Medical Oncology, Haemostaseology, Infectiology and Rheumatology, Oncologic Center Paracelsus Medical University Salzburg Austria; ^2^ Salzburg Cancer Research Institute, Center for Clinical Cancer and Immunology Trials Salzburg Austria; ^3^ Department of Surgery Paracelsus Medical University Salzburg Salzburg Austria; ^4^ Department of Sociology and Human Geography, Division of Sociology Paris Lodron University Salzburg (PLUS) Salzburg Austria; ^5^ Cancer Cluster Salzburg Salzburg Austria

**Keywords:** gemcitabine, GEMOX, nab‐paclitaxel, oxaliplatin, pancreatic cancer, treatment costs

## Abstract

**Background:**

Pancreatic cancer is mostly diagnosed in an advanced stage and treated with systemic therapy with palliative intent. Nowadays, the doublet chemotherapy of Gemcitabine and nab‐paclitaxel (Gem‐Nab) is one of the most frequently used regimens worldwide, but is not ubiquitarily available or reimbursed. Therefore, we compared the clinical efficacy of Gem‐Nab to a historical control of patients treated with gemcitabine and oxaliplatin (Gem‐Ox) at our tertiary cancer center, which was the standard treatment prior to the introduction of FOLFIRINOX.

**Methods:**

This single‐center retrospective real world study includes 121 patients diagnosed with locally advanced or primary metastatic pancreatic adenocarcinoma who were treated with chemotherapy doublet, with either Gem‐Nab or Gem‐Ox in palliative first‐line. Survival rates were analyzed using the Kaplan–Meier method, and comparisons were made with log‐rank tests. Gem‐Ox was considered as standard first line therapy at our institution for patients who were deemed fit for doublet chemotherapy between the years 2006 to 2012. These patients were compared to a cohort of patients treated with the new standard first‐line therapy of Gem‐Nab between 2013 and 2020.

**Results:**

A total of 554 patients with pancreatic cancer of all stages were screened, and 73 patients treated with Gem‐Nab and 48 patients treated with Gem‐Ox in the palliative first‐line setting were identified and included in this analysis. Patients receiving Gem‐Ox had a statistically significantly better performance score (ECOG PS) when compared to the Gem‐Nab group (Odds ratio (OR) 0.28, 95% CI 0.12–0.65, *p* = 0.005), more often suffered from locally advanced than metastatic disease (OR 3.10, 95% CI 1.27–7.91, *p* = 0.019) and were younger in age (OR 0.95, 95% CI 0.91–0.99, *p* = 0.013). Median overall survival (OS) of the whole study cohort was 10.3 months (95% CI 8.5–11.6). No statistically significant difference in OS could be observed between the Gem‐Nab and the Gem‐Ox cohort (median OS: 8.9 months (95% CI 6.4–13.5) versus 10.9 months (95% CI 9.5–13.87, *p* = 0.794, HR 1.27, 95% CI 0.85–1.91)). Median progression‐free survival (PFS) was 6.8 months in the entire cohort (95% CI 4.9–8.4). No statistically significant difference in PFS could be observed between the Gem‐Nab and the Gem‐Ox cohort (median PFS: 5.8 months (95% CI 4.3–8.2) versus 7.9 months (95% CI 5.4–9.5) *p* = 0.536, HR 1.11, 95% CI 0.74–1.67). Zero‐truncated negative binomial regressions on OS and PFS adjusting for gender, age, performance status (ECOG PS), and CA19‐9 levels yielded no significant difference between Gem‐Nab or Gem‐Ox.

**Conclusion:**

From our analysis, we could evidence no difference in outcome parameters in this retrospective analysis despite the worse prognostic pattern for GemOX. Therefore, we suggest Gem‐Ox as potential first line treatment option for inoperable locally advanced or metastatic pancreatic cancer, especially if Gem‐Nab is not available.

## Graphical abstract

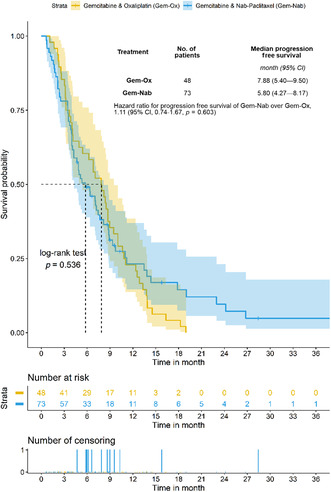

## 1 INTRODUCTION

Pancreatic cancer (PC)^1^ represents the third leading cause of cancer‐related mortality, which is predicted to further increase in coming years due to rising incidence.^2^ Progress in the development of novel drugs has been low, and prognosis is still dismal. According to Global Cancer Statistics 2020, there have been 495,773 new reported pancreatic cancer (PC) cases and 466,003 deaths from PC worldwide.^3^

PC is mostly diagnosed in an advanced stage with symptoms appearing late in the course of disease. Sensitive and specific screening examinations are missing although efforts to utilize circulating tumor DNA together with protein‐based tumor markers have been made.^4^ As a consequence, more than 80% of patients do not qualify for curative resection at initial diagnosis and therefore are treated with palliative intent.^5^

For decades, 5‐FU monotherapy was the standard of care, followed by gemcitabine in 1997 resulting in a median survival duration of 5.65 and 4.41 months for gemcitabine‐treated and 5‐FU‐treated patients, respectively (*p* = 0.0025).^6^ Compared to the former treatment standard gemcitabine, the chemotherapy doublet gemcitabine plus nab‐paclitaxel (Gem‐Nab) also demonstrated a survival benefit (median OS: 8.5 vs. 6.7 months, HR 0.72; *p* < 0.001) and a superior PFS (5.5 vs. 3.7 months, HR 0.69; *p* < 0.001), respectively, when compared to gemcitabine alone (Gem) in palliative first‐line.^7^

Based on the abovementioned positive clinical phase III trial result, the current ESMO and ASCO guidelines recommend Gem‐Nab besides modified FOLFIRINOX (mFOLFIRINOX, a triple therapy containing 5‐FU, Irinotecan, Oxaliplatin) as first‐line palliative treatment options for fit patients with advanced pancreatic cancer.^8^, ^9^

Due to fewer side effects in regard to grade 3 or 4 neutropenia, thrombocytopenia, febrile neutropenia, diarrhea and due to recommended G‐CSF usage with (m)FOLFIRINOX protocol, Gem‐Nab is currently used as first‐line therapy regimen in the majority of cases in most European countries.^10^ Furthermore, Abrams et al reported that first‐line administration of Gem‐Nab increases and is currently the most commonly used regimen in the United States since 2014.^11^ However, due to the costs of nab‐paclitaxel globally, not all patients have access to Gem‐Nab and some agencies are critical concerning financial impact on the healthcare system (e.g., NICE presents final draft guidance on nab‐ Paclitaxel for PC on September 17, 2015).

Before the advent of Gem‐Nab and mFOLFIRINOX as standard first line regimens, the combination of Gemcitabine and Oxalipatin (Gem‐Ox) was frequently employed as first‐line regimen in patients who were deemed fit for doublet chemotherapy due to its supposed superior clinical efficacy when compared to single agent Gemcitabine.

Our treatment decisions, at that time, were supported by historical results.

Louvet et al. comparing Gemcitabine monotherapy with Gem‐Ox reported patients treated with Gem‐Ox (*n* = 157) had a superior response rate compared to Gem (*n* = 156) (26.8% vs. 17.3%, respectively; *p* = 0.04), the median OS for Gem‐Ox and Gem was 9.0 and 7.1 months, respectively (*p* = 0.13) showing a trend toward clinically meaningful improvement.^12^

In 2007, Zhao et al. published the results of a study including 30 patients with advanced PC, treated with Gem‐Ox. Their findings showed an encouraging response rate (20%), suggesting that Gem‐Ox may be an effective alternative chemotherapy regimen.^13^

Furthermore, indirect evidence from the adjuvant therapy setting showed a promising 70% 1‐year relapse‐free survival with postoperative Gem‐Ox followed by Gem + Radiotherapy suggesting high efficacy.^14^

Due to these data, we frequently employed Gem‐Ox as first line palliative therapy in the years 2006 to 2012, until in 2013 Gem‐Nab became available to all our patients.

In this unicentric study, we retrospectively evaluated the clinical outcome of patients with locally advanced or primary metastatic PC treated with either Gem‐Nab (2006 to 2012) or with Gem‐Ox (2013 to 2020) as palliative first‐line chemotherapy.

## 2 METHODS

In this single‐center retrospective study, patients with pancreatic adenocarcinoma were included, who had been treated at the Department of Internal Medicine III at the Paracelsus Medical University Salzburg, Austria.

All patients over the age of 18 with pathologically proven locally advanced or primary metastatic pancreatic adenocarcinoma could be included. This retrospective analysis was approved by the Ethics Committee of the provincial government of Salzburg, Austria (415‐EP/73/795–2018).

Patient data were obtained from medical records at the Department of Internal Medicine III of the Paracelsus Medical University Salzburg, Salzburg, Austria.

We retrospectively evaluated patient characteristics, Eastern Cooperative Oncology Group (ECOG) performance score (PS),^15^ date of diagnosis, start of palliative systemic first‐line treatment, subsequent therapy protocols, PFS and OS based on review of patients' medical records, chart review, and radiology reports. OS was calculated from the date of diagnosis of primary metastatic or locally advanced PC until the day of death from any cause. The PFS was defined as the period between the start of therapy and the date of disease progression or death. Patients without any event (OS/PFS) at time of analysis were censored accordingly.

Dose modifications were made at the discretion of the treating physician. In case of chemotherapy induced peripheral neuropathy, Oxaliplatin or Nab‐Paclitaxel may have been reduced in dose or discontinued, while continuing Gemcitabine therapy alone.

As per our institutional standard treatment was continued until disease progression, unacceptable toxicities or patient wish.

Palliative first‐line therapy included Gem‐Nab (Gemcitabine 1000 mg/m^2^ and Nab‐Paclitaxel 125 mg/m^2^, respectively, on Days 1, 8, and 15 (every 4 weeks)) and Gem‐Ox (Gemcitabine 1000 mg/m^2^ and Oxaliplatin 100 mg/m^2^, respectively, on Days 1 and 8 (every 3 weeks)). Tumor markers were routinely assessed at baseline and before each treatment cycle.

### 2.1 Statistics

Data analysis for this retrospective study was descriptive in nature. OS and PFS estimates were obtained using Kaplan–Meier (KM) method, and the log‐rank test was applied to test for differences in the survival curves. The test for the proportional hazard assumption indicated a significant deviation (for OS: *x* ^2^ = 3.981, df = 1, *p* = 0.0046; for PFS: *x* ^2^ = 7.305, df = 1, *p* = 0.007). Consequently, additional statistical tests and procedures were applied to confirm the robustness against nonproportional hazard rates. Namely, the weighted Cox‐Regression was used to identify the impact of covariates on OS and PFS and zero‐truncated negative binomial regressions were applied to consider OS and PFS more strictly. A *p*‐value of <0.05 was considered to be statistically significant. All statistical analyses were performed with R 4.1.2 (R Core Team 2021).^16^

## 3 RESULTS

### 3.1 Patient characteristics

Between 2006 and 2020, 554 patients with PC were treated at Department of Internal Medicine III of the Paracelsus Medical University Salzburg and screened for inclusion into this retrospective analysis. From this cohort, 140 patients were treated with Gem‐Nab or Gem‐Ox. Patients, with upfront surgery receiving Gem‐Ox as adjuvant therapy were excluded (*n* = 19). Therefore, 121 patients with locally advanced (*n* = 26; 21%) and metastatic (*n* = 95; 79%) disease undergoing systemic treatment with palliative intent were included in our analysis (See Table 1 Consort Diagram).

**TABLE 1:**
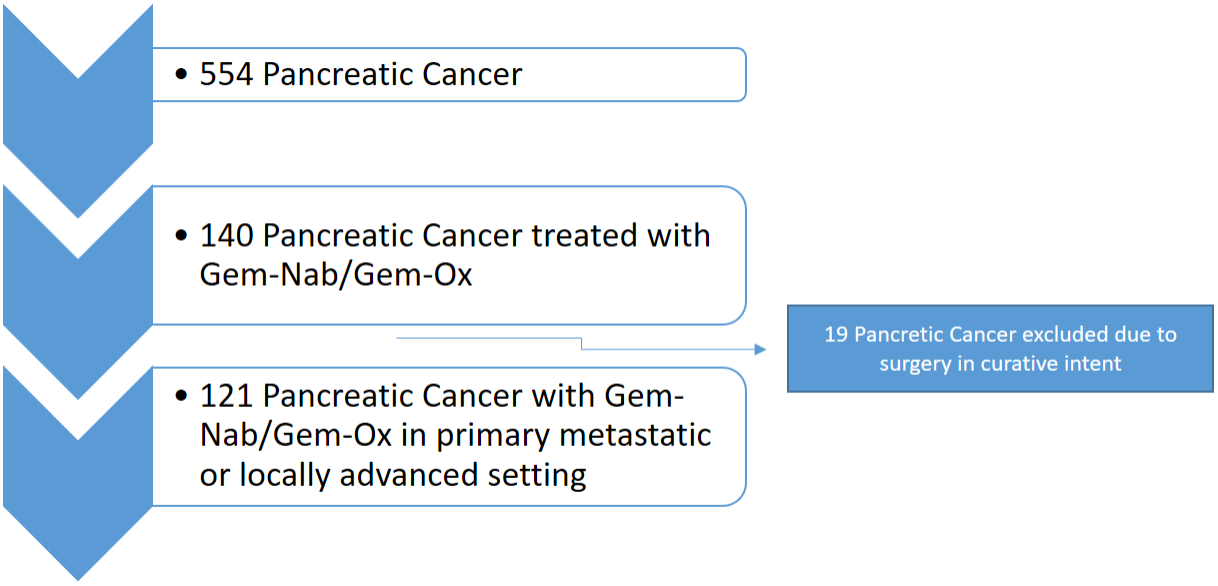
Consort diagram.

Gem‐Nab was applied to 73 patients (60%) and 48 patients (40%) received Gem‐Ox as palliative first‐line therapy. None of our patients were still on treatment at the time point of data analysis. For patient baseline characteristics, please see Table 2.

**TABLE 2:** Patient baseline characteristics.

Characteristic	Gemcitabine and Oxaliplatin, *N* = 48	Gemcitabine and Nab‐Paclitaxel, *N* = 73	Overall, *N* = 121^a^	*p*‐value^b^
Gender				0.281
Male	24 (50.0%)	45 (61.6%)	69 (57.0%)	
Female	24 (50.0%)	28 (38.4%)	52 (43.0%)	
Age at diagnosis				0.013
Mean (SD)	65 (9.35)	69 (9.11)	67 (9.41)	
Median (Range)	67 (41.00, 80)	71 (37.00, 85)	69 (37.00, 85)	
ECOG Performance status				0.005
ECOG 0 + 1	29 (64.4%)	17 (33.3%)	46 (47.9%)	
ECOG 2 + 3	16 (35.6%)	34 (66.7%)	50 (52.1%)	
CA 19.9 Median				0.259
Mean (SD)	11,902 (58,036.39)	26,111 (77,885.48)	20,331 (70,566.67)	
Median (Range)	424 (1.00, 400,000)	1155 (1.00, 413,000)	641 (1.00, 413,000)	
Subsequent systemic therapy				0.066
No	16 (33.3%)	38 (52.1%)	54 (44.6%)	
Yes	32 (66.7%)	35 (47.9%)	67 (55.4%)	
				0.019
Locally advanced	16 (33.3%)	10 (13.7%)	26 (21.5%)	
Metastatic disease	32 (66.7%)	63 (86.3%)	95 (78.5%)	

^a^ *n* (%); c (“Mean (SD)”, “Median (Range)”).

^b^ Pearson's chi‐squared test; Welch two sample *t*‐test.

Patients receiving Gem‐OX had a statistically significantly better ECOG PS compared to the Gem‐Nab group (*p* = 0.005), and were younger in age (*p* = 0.013). In the Gem‐Ox group 32 patients (66%) and in the Gem‐Nab group 35 patients (48%) received a subsequent therapy (*p* = 0.07). More patients in the Gem‐Ox group had locally advanced disease at the start of palliative front‐line therapy compared to the Gem‐Nab group (33% vs. 14%, *p* = 0.019).

Median cycle number applied for Gem‐Nab was 5.2 with applications on d1, 8, and 15 and 4,8 for Gem‐Ox (d1 and 15).

### 3.2 Survival

**FIGURE 1:**
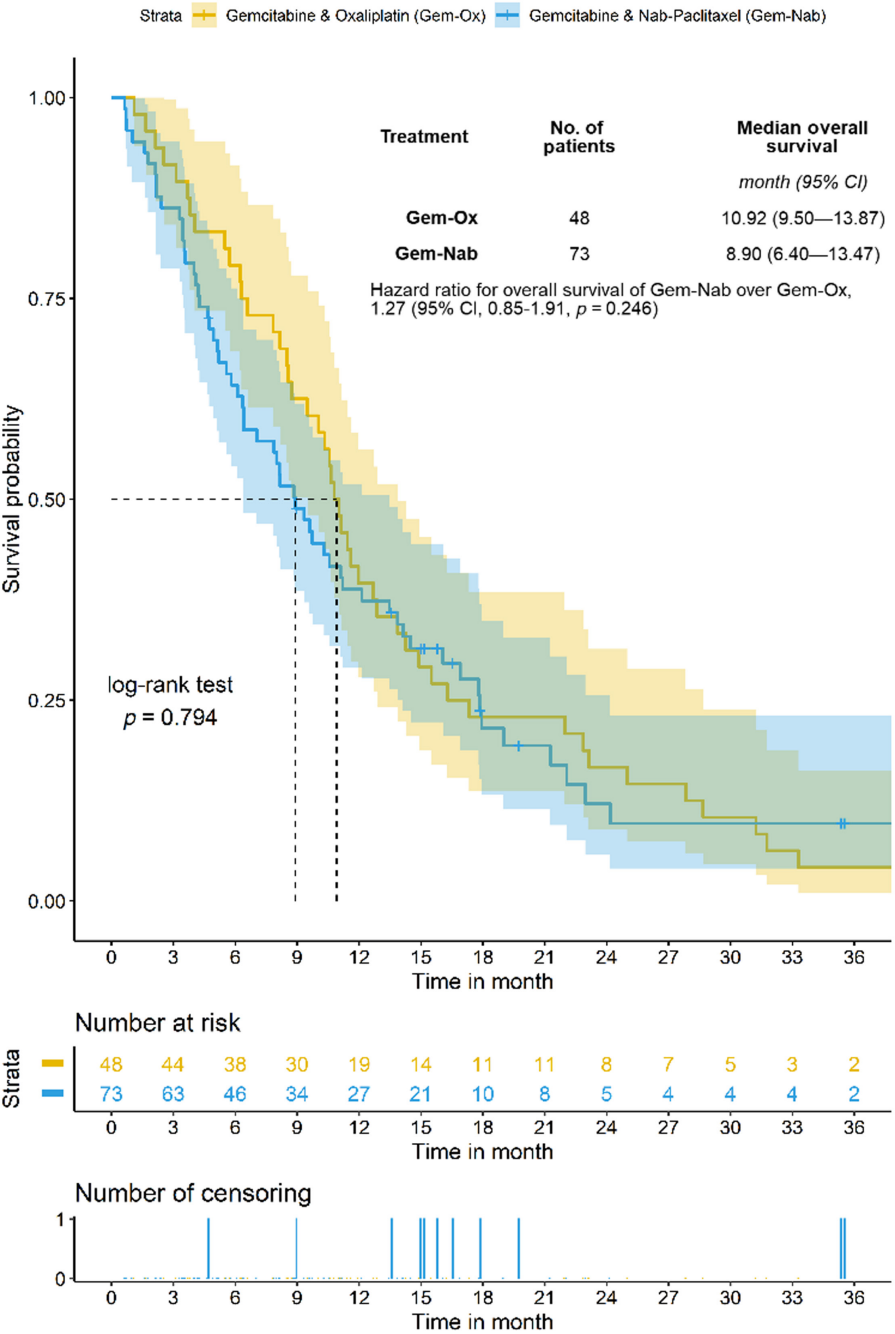
The median OS for all patients was 10.3 months. Hazard ratios are based on a weighted Cox‐regression.

**FIGURE 2:**
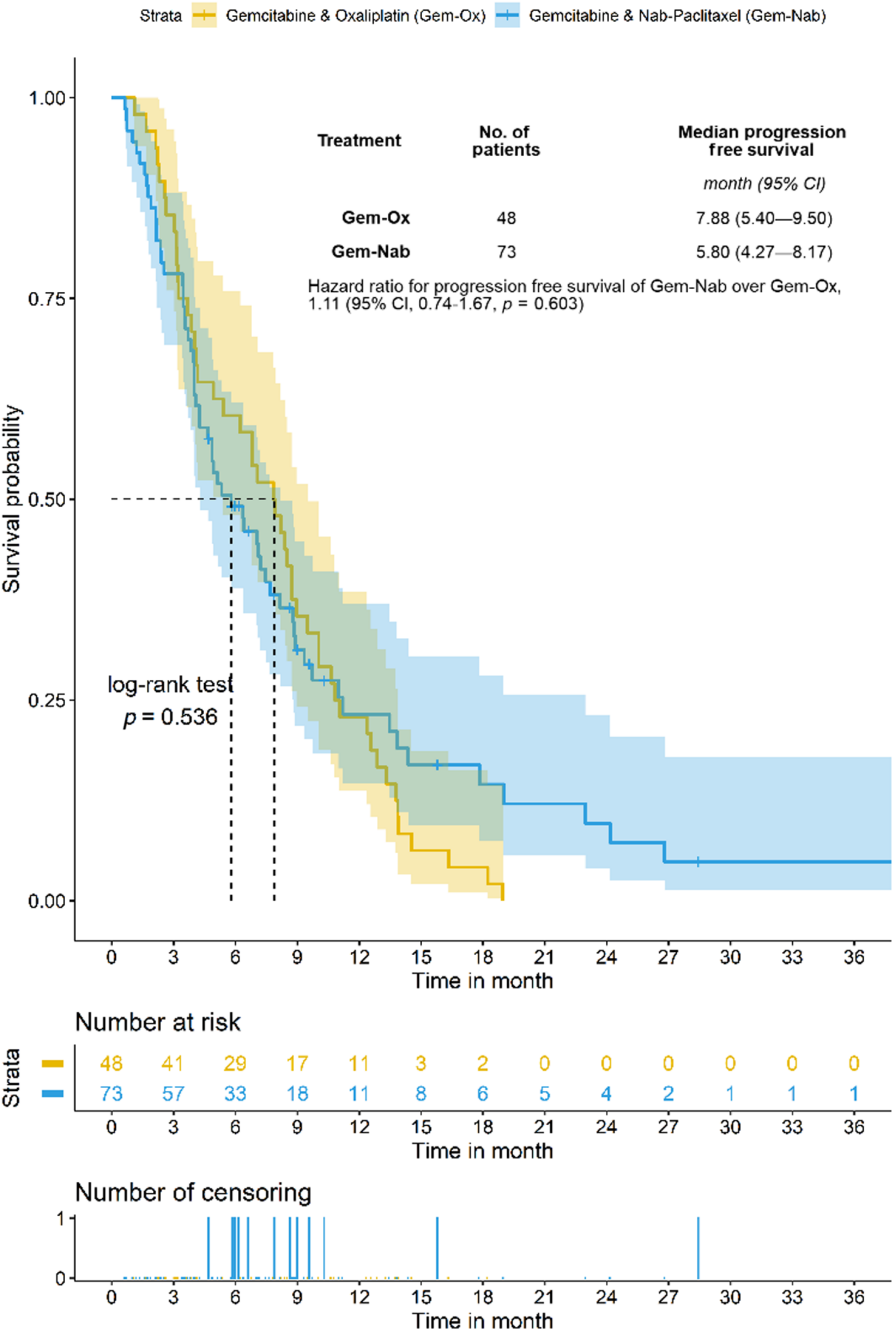
The median PFS for all patients was 8.1 months. Hazard ratios are based on a weighted Cox‐regression.

Median OS of the whole study cohort was 10.3 months (95% CI 8.5–11.6). Median OS did not statistically significantly differ between the Gem‐Nab group and the Gem‐Ox group (8.9 months vs. 10.9 months; *p* = 0.794; HR; 1.27; 95% CI 0.85 to 1.91; Figure 1). Median PFS in the entire cohort was 6.8 months (95% CI 4.9–8.4). Median PFS in the Gem‐Nab group was not superior to the Gem‐Ox group (5.8 months vs. 7.9 months; *p* = 0.536; HR, 1.11; 95% CI 0.74 to 1.67; Figure 2).

Due to the descriptive nature of the results presented so far, a weighted Cox‐regression and zero‐truncated negative binomial regression were used as robustness checks or mild sensitivity analysis (Table 3). With the weighted Cox‐regression, the nonproportional hazard rates are considered as well as covariates influencing the survival curves. The zero‐truncated negative binomial regression estimates the potential change in the survival days by the covariates. Both procedures would require more data to provide robust results, and interpretation is descriptive at most.

**TABLE 3:**
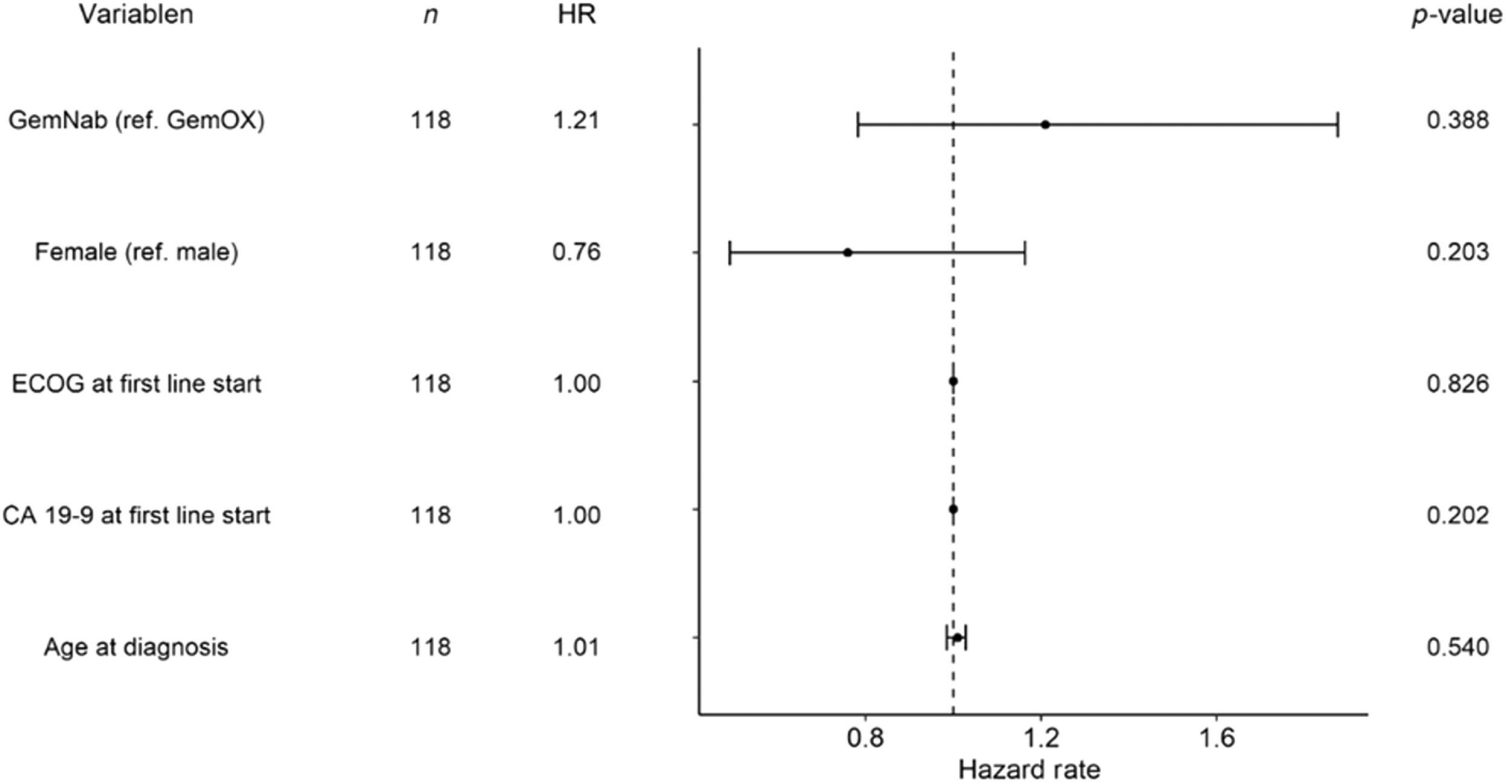
Forest plot for the estimates of the cox regression.

## 4 DISCUSSION

In our retrospective analysis, we found no evidence of superiority of Gem‐Nab over Gem‐Ox in regard to PFS (*p* = 0.536; HR: 1.11; Figure 2) or OS (*p* = 0.794; HR: 1.27; Figure 1).

In the Gem‐Nab arm, there were more patients with a worse ECOG performance status and older age compared to the Gem‐Ox group. Furthermore, locally advanced disease at treatment start with palliative intent was more frequently found in the Gem‐Ox group, and we observed a trend that fewer patients in the Gem‐Nab arm compared to the Gem‐Ox group received a subsequent systemic therapy. We cannot rule out a potential bias of the abovementioned findings in favor of the Gem‐Ox group in regard to clinical outcome. However, Gem‐Ox was given in the time period from 2006 to 2012, when second‐line treatment with 5‐Fluorouracil and nanoliposomal irinotecan was not yet known or available. In contrast hereto, patients in the Gem‐Nab group were treated between 2013 and 2020, 20% of whom received 5‐Fluorouracil and nanoliposomal irinotecan as subsequent therapy. Furthermore, the advancements of supportive care over time are thought to positively impact survival of cancer patients in general.^17^

Polychemotherapy, like Gem‐Nab, is the current standard of care and recommended by ASCO, NCCN, and ESMO guidelines based on positive Phase III trial results, for fit patients with advanced pancreatic cancer as it significantly increases survival compared to monotherapy with gemcitabine and due to its potential to maintain quality of life.^7^, ^18^ Gem‐Ox, however, is not endorsed by NCCN guidelines.

Our results are in line with the literature. A Chinese single center experience concerning outcome of (m)FOLFIRINOX, Gem‐Nab and Gem‐Ox in unresectable pancreatic cancer was reported: In this analysis, the median OS times were 11.1, 10.1 and 10.2 months (*p* = 0.75) in the Gem‐Nab, mFOLFIRINOX and Gem‐Ox cohort, respectively.^19^ Furthermore, Louvet et al. reported a median OS for Gem‐Ox of 9.0 months.^12^

### 4.1 Limitations of this study

The *first limitation* of this analysis is its retrospective and unicentric nature. *Secondly*, patient numbers in each treatment arm can be regarded as rather low constraining inferential statistics. *Third*, the hazard ratios of both treatment groups are nonproportional implying limitations in the graphical interpretation of the Kaplan–Meier curves. We applied weighted Cox‐Regression and zero‐truncated negative binomial regression to validate the findings from the analysis of the Kaplan–Meier curves. Although we are confident that our results support our main statistical claim of only minor differences between Gem‐Ox and Gem‐Nab treatment, the results need to be interpreted with caution and we urge to consider the reported results as preliminary findings. Ideally, our findings have to be further validated in a prospective trial including a larger cohort.

### 4.2 *Novelty* of results

Summarizing the key finding of the results, Gem‐Nab and Gem‐Ox treatment show only minor differences in overall survival and progression‐free survival time. In most models, the differences are not statistically significant, and therefore, it seems plausible to consider both treatments as similar effective. In our opinion, these reported data are of clinical significance especially for patients not having access to novel chemotherapeutic agents like nab‐paclitaxel. Oxaliplatin is a valid combination with Gem in this setting in order to guarantee application of a chemotherapy doublet and increasing response rates and survival.

## AUTHOR CONTRIBUTIONS

**Konstantin Schlick:** Conceptualization (equal); project administration (equal); writing – original draft (equal). **Antonia Gantschnigg:** Conceptualization (equal); resources (equal); visualization (equal). **Alexander Seymer:** Data curation (equal); formal analysis (equal); methodology (equal); writing – review and editing (equal). **Florian Huemer:** Data curation (equal); writing – review and editing (equal). **Richard Greil:** Conceptualization (equal); investigation (equal); writing – review and editing (equal). **Lukas Weiss:** Supervision (equal); writing – review and editing (equal).

## FUNDING INFORMATION

The authors received a partial financial support by SERVIER Austria GmbH for publication of this article.

## CONFLICT OF INTEREST STATEMENT

The authors declared no potential conflicts of interest with respect to the research, authorship, and/or publication of this article.

## ETHICS STATEMENT

The analysis was approved by the Salzburg Ethics Commission (EP/73/789) and informed consent was waived by the decision of the same institution.

## Supporting information


Data S1

## Data Availability

All data supporting our findings are available from corresponding author.
