# Supplementary material for: Comparison of gemcitabine plus oxaliplatin versus gemcitabine plus nab‐paclitaxel as first‐line chemotherapy for advanced pancreatic adenocarcinoma: A single‐center retrospective analysis
Source: Cancer Med. 2023 Aug 3;12(16):16997–7004. doi: 10.1002/cam4.6334 (PMC10501299; doi:10.1002/cam4.6334)
Supplement: Supplementary file 1 — Data S1 [file CAM4-12-16997-s001.docx]

Supplementary Tables and Figures

S.Table 1: Zero-Truncated Negative Binomial Regression on Overall Survival

|  | **Marginal Effects** | **Incidence Rate Ratio** |
| --- | --- | --- |
| **Gem-Nab (ref. Gem-Ox)** | -82.148 (59.798) | 0.802 (0.125) |
|  | 0.170 (-200.630, 36.334) | 0.157 (0.590, 1.085) |
| **Female (ref. male)** | 26.156 (55.337) | 1.074 (0.162) |
|  | 0.636 (-83.488, 135.800) | 0.635 (0.798, 1.451) |
| **Age at diagnosis** | 1.751 (2.907) | 1.005 (0.008) |
|  | 0.547 (-4.008, 7.510) | 0.546 (0.989, 1.020) |
| **ECOG at first line start** | -0.007 (0.012) | 1.000 (0.000) |
|  | 0.576 (-0.031, 0.017) | 0.576 (1.000, 1.000) |
| **CA 19-9 at first line start** | 0.000 (0.000) | 1.000 (0.000) |
|  | 0.240 (-0.001, 0.000) | 0.238 (1.000, 1.000) |
| **(Intercept)** |  | 300.649 (162.718)*** |
|  |  | 0.000 (105.659, 915.002) |
| **Num.Obs.** | 118 | 118 |
| **AIC** | 1628.9 | 1628.9 |
| **BIC** | 1648.3 | 1648.3 |
| **Log.Lik.** | -807.460 |  |
| **RMSE** |  | 304.63 |
| Note: Incidence rate ratios are interpretable like odds ratios. E.g. the incidence rate of 1.074 for females means that females have a 1.074 better chance for a longer overall survival. The marginal effect is the effect size in days of overall survival. E.g. females would survive 26.156 days longer than men. All coefficients are not significant. | | |

S.Table 2: Zero-Truncated Negative Binomial Regression on Progression Free Survival Days

|  | **Marginal effects** | **Incidence rate ratio** |
| --- | --- | --- |
| **Gem-Nab (ref. GemOx)** | -22.344 (32.503) | 0.907 (0.128) |
|  | 0.492 (-86.745, 42.057) | 0.487 (0.685, 1.195) |
| **Female (ref. male)** | 48.178 (31.841) | 1.234 (0.168) |
|  | 0.130 (-14.910, 111.267) | 0.122 (0.944, 1.619) |
| **Age at diagnosis** | 3.802 (1.649)* | 1.017 (0.007)* |
|  | 0.021 (0.535, 7.069) | 0.020 (1.002, 1.031) |
| **ECOG at first line start** | -0.001 (0.007) | 1.000 (0.000) |
|  | 0.858 (-0.015, 0.012) | 0.858 (1.000, 1.000) |
| **CA 19-9 at first line start** | 0.000 (0.000) | 1.000 (0.000) |
|  | 0.191 (-0.001, 0.000) | 0.190 (1.000, 1.000) |
| **(Intercept)** |  | 71.967 (35.281)*** |
|  |  | 0.000 (28.298, 193.450) |
| **Num.Obs.** | 118 | 118 |
| **AIC** | 1504.2 | 1504.2 |
| **BIC** | 1523.6 | 1523.6 |
| **Log.Lik.** | -745.119 |  |
| **RMSE** |  | 180.75 |
| Note: Incidence rate ratios are interpretable like odds ratios. E.g. the incidence rate of 1.234 for females means that females have a 1.234 better chance for a longer progression free survival. The marginal effect is the effect size in days of progression free survival. E.g. females would survive progression free 48.178 days longer than men. All coefficients are not significant. | | |
